# Inferior Spear-like Lens Opacity as a Sign of Keratoconus

**DOI:** 10.18502/jovr.v17i1.10165

**Published:** 2022-01-21

**Authors:** Ramin Salouti, Amir Khosravi, Majid Fardaei, Mohammad Zamani, Mahmoud Nejabat, Maryam Ghoreyshi, Mahboobeh Yazdanpanah, Kia Salouti, M. Hossein Nowroozzadeh

**Affiliations:** ^1^Department of Ophthalmology, School of Medicine, Shiraz University of Medical Sciences, Shiraz, Iran; ^2^Salouti Cornea Research Center, Salouti Eye Clinic, Shiraz, Iran; ^3^Department of Medical Genetics, Shiraz University of Medical Sciences, Shiraz, Iran; ^4^Health Policy Research Center, Shiraz University of Medical Sciences, Shiraz, Iran; ^5^Science Department, The university of British Columbia, Vancouver, Canada

**Keywords:** Cataract, Feather-shape, Keratoconus, Lens Opacity, Sectoral

## Abstract

**Purpose:**

To report 21 cases of typical inferior feather-shape lens opacity associated with keratoconus.

**Methods:**

In this cross-sectional study, we evaluated the association of keratoconus with inferior feather-shape lens opacity in refractive surgery candidates. Visual acuity, demographic, refractive, and topographic characteristics of 26 eyes of 21 patients with inferior feather-shape lens opacity were evaluated in detail. Pedigree analysis was also performed to assess possible inheritance.

**Results:**

Overall, 2122 out of 33,368 cases (6.4%) without lens opacity had keratoconus, while 20 out of 21 patients (95.2%) with peculiar lens opacity had definite keratoconus (*P* < 0.001). Lens opacity was bilateral in 5 cases (24%), and keratoconus was bilateral in all 20 patients with lens opacity. Nine eyes out of thirty-six with a complete data record (25%) had a severe keratoconus and underwent deep lamellar keratoplasty, while 11 (31%) had forme fruste keratoconus. Pedigrees were drawn for eight patients, most families of whom suggested an X-linked recessive inheritance.

**Conclusion:**

The present study was the first to investigate patients with a peculiar inferior feather-shape lens opacity accompanied by bilateral keratoconus, which was observed in 95% of the patients. This finding should raise awareness as to the possibility of diagnosing keratoconus in the eyes of the patients with these characteristics.

##  INTRODUCTION

Keratoconus is a noninflammatory, often bilateral, and asymmetric corneal disorder^[1]^ in which the progressive central or paracentral corneal thinning leads to the bulging of the thinned cornea.^[2]^ Keratoconus often occurs as an isolated disorder.^[3,4]^ Despite the extensive studies carried out over the recent decades on the etiology and pathogenesis of keratoconus, the exact pathophysiology and biochemical mechanisms of keratoconus is yet to be fully understood. However, several environmental (such as eye rubbing, atopy, sun exposure, and geography), genetic (i.e., familial inheritance, association with other known genetic disorders, and predisposing mutations), and biomechanical mechanisms have been proposed to contribute to this ectatic disorder.^[5]^ Investigating the possible association of keratoconus with other diseases requires understanding of the pathogenesis and biochemical mechanisms of this condition.

Since keratoconus is a progressive disorder, an early diagnosis to save uncorrected visual performance is of utmost importance. Unfortunately, certain detection of early keratoconus in refractive surgery candidates is impossible, even with the help of the latest corneal imaging technologies. Extra-corneal clues such as genetic susceptibility might help to spot early keratoconus in uncertain situations. In our current study, we report 21 patients with an inferior feather-shape lens opacity accompanied by bilateral keratoconus at different stages in almost all of the cases.

##  METHODS

From January 2010 to July 2017, all refractive surgery candidates who were referred to *Salouti Eye Clinic *were evaluated for inferior feather-shape lens opacity by using slit-lamp examinations with dilated pupils. Pentacam HR (Oculus Optikgeräte GmbH) was also obtained for all of these patients, where any grade of keratoconus could be detected.

We also collected and analyzed all relevant data from these 21 patients who were diagnosed with typical feather-shape lens opacity. They underwent a complete ophthalmic examination including visual acuity, refraction, slit-lamp biomicroscopy, and fundus examination. The recorded data comprised of the corrected distance visual acuity, spherical equivalent refraction, Pentacam keratometric, pachymetric and keratoconus indices (Topographic Keratoconus Classification [TKC]). The Pentacam keratoconus indices consisted of the index of surface variance (ISV), index of vertical asymmetry (IVA), keratoconus index (KI), center keratoconus index (CKI), index of height asymmetry (IHA), index of height decentration (IHD), and minimum sagittal curvature (Rmin).

Clinical evaluations were followed by a detailed interview, which included questions about demographics, the assessment of the presence or absence of other diseases, drug intake, etc. Pedigrees were drawn up to three generations in order to evaluate the keratoconus mode of inheritance. Informed consent was obtained from the 21 participants who had cataract. The study protocol was approved by the Ethics Committee at Shiraz University of Medical Sciences and adhered to the principles of the Declaration of Helsinki.

All statistical analyses were performed using SPSS for Windows (Version 22 SPSS Inc., Chicago, IL, USA). Numerical variables were described as median (minimum to maximum).

##  RESULTS

We assessed the medical charts of 33,389 refractive surgery candidates, of whom 21 patients had the typical feather-shape lens opacity. Overall, 2122 out of 33,368 cases (6.4%) without lens opacity had keratoconus, while 20 out of 21 patients (95.2%) with lens opacity had definite keratoconus (*P* < 0.001).

**Table 1:** Demographic, refractive, and keratometric characteristics of cases


Case	**Age**	**Sex**	**Keratoconus in family**	**Eye**	**SE**	**CDVA**	**K ${}_{\mathrm{mean}}$ **	**K ${}_{\max}$ **	**K ${}_{\mathrm{astig}}$ **	**P ${}_{\min}$ **
	**(yr)**				**(D)**		**(D)**	**(D)**	**(D)**	**(µm)**
1	24	M	Cousin	OD	–6.25	20/25	49.7	54.5	3.1	452
				OS	–6.5	20/30	49.7	55.9	3.6	422
2	16	M	Cousin	OD	–3.75	20/200	48	53.9	4.2	459
				OS	–9.75	20/60	44.9	51.2	2.8	572
3	25	M	Sister; Cousin; Two first cousins once removed	OD	–2.25	20/40	46.8	49.5	2.1	516
				OS	–5.75	20/200	51.1	55.3	2.8	498
4	52	M	–	OD	–9	20/100	45.3	53.9	4.2	558
				OS	–0.5	20/50	45.5	53.9	4	545
5	13	M	–	OD	–1.5	20/20	47.7	54.6	2	517
				OS	–1	20/25	47.9	53.2	1.9	451
6	26	M	–	OD	–3	20/20	46.8	57.6	7.6	478
				OS	–0.5	20/200	54.2	59.5	6.6	475
7	33	M	Two uncles	OD	–3.75	20/50	42.9	50.7	4.2	687
				OS	–1.25	20/50	49.3	52.1	1.3	410
8	24	M	–	OD	–2.5	20/30	51.2	55.2	3.2	391
				OS	–1.5	20/30	52	55.9	3	362
9	32	M	Sister	OD	–3.75	20/40	42.6	50.1	8.2	541
				OS	–10	20/60	48.8	58.5	5.7	495
10	29	F	–	OD	–5.25	20/60	49.4	63.9	3.9	548
				OS	–4	20/40	44.3	52	4.5	492
11	27	F	Sister	OD	–4	20/30	44.1	45.3	1.4	530
				OS	–3	20/30	43.5	45.5	2	543
12	29	M	Cousin	OD	–1.5	20/20	44.1	45.3	1.4	530
				OS	–0.5	20/20	47.3	56.1	3.6	523
13	43	M	–	OD	–3.25	20/40	47.4	48.4	0.9	530
				OS	–2.75	20/30	47.8	48.6	1	522
14	26	M	Cousin	OD	–3.25	20/40	45.3	50	1.7	519
				OS	–2.75	20/30	45.2	48.3	0.7	516
15	35	F	Mother and brother	OD	–3.25	20/40	45.7	48.5	3.3	486
				OS	–6.75	20/50	47.6	48.5	4.2	480
16	37	M	–	OD	–9.25	20/50	47.8	54	3.5	455
				OS	–9.75	20/50	47.9	54	4.8	449
17	27	F	–	OD	–11.25	20/50	52	54.6	4.1	397
				OS	–11.25	20/40	51.9	54.6	3.3	408
18	28	M	–	OD	–13.25	20/80	53.4	55.2	3.7	445
				OS	–13	20/80	55.1	55.2	2.9	421
CDVA, corrected distance visual acuity; D, diopter; F, female; K ${}_{\mathrm{astig}}$ , astigmatic keratometry; K ${}_{\mathrm{mean}}$ , mean keratometry; K ${}_{\max}$ , maximum keratometry; M, male; OD, right eye; OS, left eye; P ${}_{\min}$ , minimum pachymetry; Y, year.										

**Table 2:** Pentacam keratoconus indices and feather-shape lens opacity in reported cases


Case	**Eye**	**Lens opacity**	TKC*	**ISV ${}^{†}$ **	**IVA ${}^{†}$ **	**KI ${}^{†}$ **	**CKI ${}^{†}$ **	**IHA ${}^{†}$ **	**IHD ${}^{†}$ **	**R ${}_{\min}$ ${}^{†}$ **
1	OD	+	1	50	0.38	1.08	1.02	33.8	0.048	6.2
	OS		1	60	0.56	1.02	*1.03*	40.1	0.066	6.03
2	OD	+	2–3	98	1.15	1.3	1.04	27.2	0.089	6.27
	OS		Post-graft	50	0.45	0.92	0.97	5.2	0.047	6.59
3	OD		1	44	0.46	1.1	*1.03*	15.3	0.028	6.82
	OS	+	2	83	0.82	1.16	1.07	15.6	0.06	6.11
4	OD	+	Post-graft	57	0.42	0.88	0.96	12.1	0.054	6.26
	OS		Post-graft	57	0.42	0.88	0.96	12.1	0.054	6.26
5	OD		Post-graft	48	0.43	1.08	0.97	26	0.038	6.19
	OS	+	2–3	94	1.12	1.24	1.05	4.6	0.103	6.34
6	OD	+	2	76	0.64	1.09	0.97	37.6	0.069	5.86
	OS	+	Post-graft	80	0.8	0.88	1.01	*20.1*	0.125	5.67
7	OD		Post-graft	47	*0.31*	0.96	0.96	9.9	0.024	6.65
	OS	+	2	74	0.74	1.23	1.04	5.9	0.05	6.47
8	OD	+	2	76	0.77	1.24	1.05	1	0.076	6.12
	OS	+	2	83	0.81	1.24	1.05	2.6	0.078	6.03
9	OD		1	52	0.24	0.94	0.99	16.4	0.017	6.73
	OS	+	1	63	0.55	1.09	0.97	7.3	0.047	5.76
10	OD	+	Post-graft	18	0.1	1.08	0.97	26	0.038	6.2
	OS		1	44	0.5	1.1	*1.03*	15.1	0.028	6.82
11 ${}^{‡}$	OD	+	0	14	0.03	1.01	1.01	0.2	0.001	7.45
	OS		0	18	0.07	1.02	1.01	5.1	0.005	7.42
12	OD		Post-graft	14	0.03	1.01	1.01	0.2	0.001	7.45
	OS	+	Post-graft	51	0.25	0.97	0.97	23.5	0.024	6.02
13	OD		1	23	0.12	1.04	1.01	5.5	0.01	6.97
	OS	+	1	24	0.13	1.09	1.01	5.6	0.01	6.95
14	OD	+	1–2	44	0.5	1.1	1.03	15.1	0.028	6.82
	OS		1	45	0.52	1.3	1.03	16.3	0.032	6.7
15	OD	+	1	42	0.47	1.1	1.01	25.6	0.035	6.78
	OS	+	2	60	0.66	1.15	1.04	21	0.05	6.48
16	OD		1	42	*0.31*	1.03	1.02	26.8	0.05	6.57
	OS	+	1–2	51	0.45	1.09	1.02	*20*	0.055	6.49
17	OD	+	2	77	0.61	1.25	1.06	34	0.053	5.99
	OS	+	2–3	87	0.76	1.3	1.06	21	0.058	6.08
18	OD	+	3	115	1.1	1.31	1.12	61.1	0.124	5.56
	OS	+	3	98	0.76	1.24	1.1	35	0.081	5.6
*The cases that are marked as “Post-graft” are those that had undergone lamellar keratoplasty for advanced keratoconus ${}^{†}$ The typographical coding of Pentacam keratoconus indices are based on the Pentacam definition, in which plain text refers to normal index, underlined letters to abnormal, and *italic* letters to suspicious values ${}^{‡}$ This case had typical cataract but no sign of keratoconusCKI, center keratoconus index; IHA, index of height asymmetry; IHD, index of height decentration; ISV, index of surface variance; IVA, index of vertical asymmetry; KI, keratoconus index; R ${}_{\min}$ , minimum sagittal curvature; TKC, topographic keratoconus classification										

**Figure 1:**
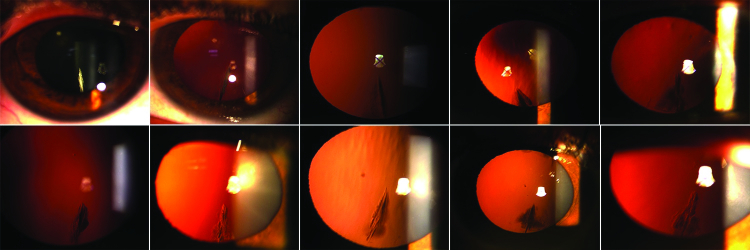
The typical inferior feather-shape lens opacity in 10 eyes of 10 cases from the cohort.

**Figure 2:**
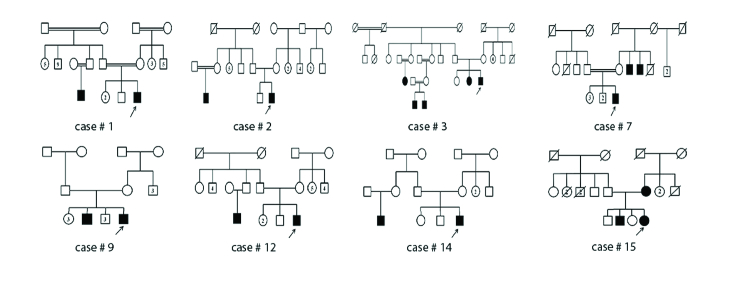
The pedigree drawings of eight cases with combined inferior feather-shape lens opacity and keratoconus. The number assigned to each case corresponds to the patients' numbers provided in Tables 1 and 2.

All lens opacities were inferior sectoral and triangular in shape, with bases toward the equator. Although the width of the opacities varied among the patients, most had a width of about one clock hour or less. Densities of the opacities were also different among the patients, some of whom had a central dense line. The borders of the cataracts were fluffy, and most of them resembled a feather [Figure 1]. The opacities were bilateral in 5 cases (24%), and keratoconus was bilateral in all 20 cases who had the ectatic disorder. No patients had Down syndrome, atopic disease, or history of corticosteroid use. Nine out of thirty-six eyes with complete data (25%) recorded had a severe keratoconus and underwent deep lamellar keratoplasty, and 11 (31%) had forme fruste keratoconus (grade 1 in Pentacam). All diagnoses of combined lens-opacity/keratoconus were made before any intervention for treating keratoconus. Only one female patient with this characteristic form of lens opacity had no corneal finding in favor of keratoconus in her prior frequent examinations. However, her first-degree relatives had a positive history of keratoc. The other three patients did not agree to be interviewed/re-examined, so their detailed data are unavailable.

As a result, a total of 18 patients, 14 males and 4 females, were evaluated in detail. The median age was 27.5 years, ranging from 13 to 52 years. The median of the visual acuities of the patients with combined keratoconus/lens-opacity was 20/50 ranging from 20/20 to 20/200. The median of patient's spherical equivalent refraction was –4.00 D (range, –13.25 to –0.50). The medians (ranges) of the keratometric and pachymetric indices were as follows: mean keratometry, 48.8 D (44.1 to 55.1); maximum keratometry, 54.6 D (45.3 to 63.9); astigmatic keratometry, 3.3 D (1.0 to 7.6); and thinnest point, 475 µm (362 to 558). Tables 1 and 2 summarize the patients' demographic, refractive, and topographic information. Of the nine eyes with more advanced keratoconus that had undergone the graft procedure, eight belonged to men (28.6% of male's eyes) and only one to a woman (12.5% of female's eyes), suggesting a more severe phenotype in males. This is further corroborated by the lower median of TKC in females as compared to males (1.5 vs 2.0). However, due to the limited number of patients, we were not able to perform a complete statistical analysis on the patients.

None of the 18 patients interviewed declared any family relationship with one another. According to the information accessed, eight patients reported to have at least one other person with keratoconus among their family members [Table 1]. We were able to draw the pedigrees of all these patients, which were more suggestive of an X-linked recessive inheritance in most cases [Figure 2]. As an ongoing project, we will try to elucidate the genetic basis of combined keratoconus/feather-shape lens opacity in future studies.

##  DISCUSSION

In this study, we introduced a novel association between inferior feather-shape lens opacities and keratoconus. This type of lens opacity is more easily visualized when the pupils are dilated; however, they could still be detected in most eyes with undilated pupils.

Keratoconus is a multifactorial disorder, originating from complex interaction between many genetic and environmental factors. In this regard, previous studies have reported that 0.5 to 15.0% of individuals with Down syndrome are also affected by keratoconus. This association is 10–300 times higher as compared with a normal population.^[6,7,8]^ It has been suggested that the primary cause of this association is eye rubbing due to the increased rate of blepharitis observed in about 46% of patients with Down syndrome.^[8]^ Other studies have reported a higher incidence or a more severe form of keratoconus in subjects with vernal keratoconjunctivitis.^[9,10]^ About 40% of patients with Leber's congenital amaurosis are affected by keratoconus;^[11,12]^ genetic factors and eye rubbing have been suggested as possible mechanisms.^[12]^ There are also possible associations reported between keratoconus and advanced mitral valve prolapse,^[13,14]^ and certain connective tissue disorders such as Osteogenesis Imperfecta^[15]^ and Ehlers-Danlos syndrome subtype VI.^[16]^

A few studies have found this association between keratoconus and lens opacities. A family affected with keratoconus and anterior polar cataract was reported back in 1931.^[17]^ The same phenotype with a supposed genetic association was reported in a large Northern Irish family.^[18,19,20]^ Co-incidence of cataract and keratoconus has also been reported in patients with atopic dermatitis.^[21,22]^

This is the first study to report a combination of keratoconus with a peculiar feather-shape lens opacity in a series of patients. We are not sure about the pattern of inheritance; however, the absence of male-to-male transmission, the lower number of affected females, and the milder phenotype in females are mostly suggestive of an X-linked inheritance in most pedigrees. In addition, the penetrance of the lens opacity was varied in the keratoconus cases of the index patients' families. Intuitively, the inferior location of both the lens opacity and the keratoconus cone might suggest a developmental error in the early fetal development of the eye, where the lens and cornea must be precisely separated from each other. Since some cases with bilateral keratoconus (which is usually asymmetric) have unilateral feather-shape lens opacity, the proposed developmental error might present in different severities with resultant phenotypes of various origins. It is logical to suppose that the keratoconus and the related lens opacity have the same genetic basis but with different degrees of penetrance and expressivity. In other words, the background lens abnormality probably involves both eyes; however, the visible lens opacity may be present in one or both eyes. Whether or not there exists a single gene basis for such an association is the subject of our future investigation.

Among the limitations of our study, mention can be made of the retrospective nature in the primary collection of data for all of the cases. However, all patients who were willing to participate were re-examined and interviewed prospectively, and the nature of the findings was not subject to classic biases posed by retrospective design (such as inadequate recording, misclassification, etc.). In addition, although we amassed detailed information on our patients, there was an inability to exact genetic evidence for the observed association, which would be the subject of future investigation for this cohort of patients.

In summary, our study revealed that the typical inferior feather-shape lens opacity is suggestive of an associated keratoconus, particularly in cases who might not show the clinical signs of keratoconus. Therefore, we recommend an appropriate workup to diagnose keratoconus in patients with such type of cataract.

##  Financial Support and Sponsorship

None.

##  Conflicts of Interest

None declared.
